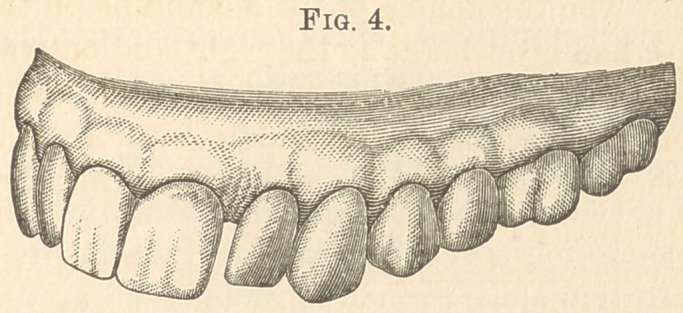# Pyorrhœa Alveolaris

**Published:** 1889-12

**Authors:** George S. Allan

**Affiliations:** New York City


### PYORRHŒA ALVEOLARIS.[1]

BY GEORGE S. ALLAN, D.D.S., NEW YORK CITY.

In reply to your kind invitation to read a paper on pyorrhœa alveolaris, I wish to present such thoughts and principles to you as have in the main guided me in my practice, and not a rehash of clippings and theories from text-books and the current literature. On one or two minor points only do I claim anything original. It would be difficult, indeed, to go over so well beaten a track and find much that was new, or garner much where so many reapers have gone before. Doubtless, as I go on, many of you will recognize the theories and principles of the authors you are most familiar with, and will give them their proper credit. I do not wish or intend to make the slightest effort to steal their thunder. I give them full credit, and gratefully acknowledge my indebtedness. One name only will I refer to, and that one I could not well omit; I owe him far more than all others combined, for he has given us the most complete, concise, and philosophical papers on this as well as kindred topics that we possess. I refer, of course, to Dr. Black, of the Chicago College. Until he took the subject in hand and whipped it into shape, we had nothing but a medley of crude theories, and still cruder methods of practice. Now, all is changed, and we can walk rapidly over a road well paved with facts and lighted with principles. I gladly take this opportunity to render thanks where thanks are so well due.

It seems very strange to many who have given attention to the subject, and have such cases as we are to consider constantly before them, how little the profession in general is interested in them and how universally they are put to one side,—neglected or avoided altogether. Nine cases out of ten, at least, that come into my hands have the same story to tell of how they had thought a cure, or even a relief, was impossible, for their dentists have told them "there was no hope; the trouble being constitutional, in their blood, and nothing could be done." More professional crimes are committed in this department of our practice than in any other I know of, for I hold it is a crime for a dentist, to whose professional skill and care

---

[1] Read before the New Jersey State Dental Society, at its nineteenth annual session at Asbury Park, July 18, 1889.

a patient commits himself, to offer him ignorance where he expects knowledge, and mislead him as to his chances and opportunities for a cure. Still less is it excusable for a dentist to allow, in any case, his patient to acquire the disease while he is in his care. If my views are well founded, its beginnings are always capable of being easily detected and as easily warded off. I would be heartily ashamed of myself if a patient of mine acquired it during the time his or her teeth were in my charge, unless it was through such neglect on their part as would make my directions and work on their teeth useless for warding it off. I cannot lay too great stress on this point, and repeat my assertion that the blame rests on the dentist in all such cases.

I have taken some pains to accumulate statistics as to the relative numbers and importance of the teeth lost in this way as compared with those lost by decay. Of course, they are incomplete and only relatively valuable, still they are impressive, and should wake us up to the importance of the subject and of our duty in studying it in all its phases. I will give you the net results only of my investigations. 1st. The actual number of all teeth lost by pyorrhœa alveolaris (of course, I leave out of this estimate the deciduous teeth) approximates very closely, if it does not actually reach, the number lost by decay (caries). 2d. The molars and bicuspids are affected by it, as compared with the front teeth, in the proportion of two or three of the former to one of the latter. 3d. Middle and old age are specially liable to it, and hardly at all to decay.

As to the difficulties attending treatment. In its earlier stages success is as certain as in that of decay and generally as lasting. In its later stages it is more difficult and far from as promising in good results. The difference is the same in degree that a builder would have in saving a house from destruction whose foundation was gone or badly impaired, or only some of the upper stories out of order.

As I propose, as before intimated, to consider the subject from a personal stand-point, and give you only such thoughts and ideas as naturally present themselves from my own experience, it may be well here to outline, in brief, what I consider to be the characteristics or salient features of the disease, so that you may the more easily recognize it. For convenience' sake I will group the symptoms under two headings,—the manifest and the obscure. Under the first heading will belong those that the patient takes painful cognizance of, and most frequently brings him to your chair; and under the second, those which it requires the educated eye and touch of the dentist to detect and point out. The former belong to the later stages of the disease, the latter to the earlier.

First and most prominent of the former class of symptoms is the loosening of the teeth. This may or may not be accompanied by a recession of the gums, but most frequently it is. It may come on gradually and unaccompanied by pain, or it may make most rapid progress and cause more or less soreness and inconvenience. A careless patient would have his attention first drawn to the trouble by noticing that one or more teeth felt lame or were tender to the touch, or when eating, but at other times were quiet and peaceful and gave no annoyance. Then on placing one of his fingers, as he naturally would, on the offending member, he would find it had lost some of its firmness and was shaky in its socket.

When, as frequently happens, recession of the gums is the most prominent feature, it will be noticed that on one face of the tooth the root is unduly exposed, the gum having disappeared, but the tooth need not of necessity feel loose, being held in place by healthy tissues on the remaining portions, and may be still further steadied by its neighbors on either side. This we would call a second symptom, and a third symptom appears in a softened, tumefied gum, one that bleeds easily and has a dark purplish color. A fourth symptom would be a bad breath and a disagreeable taste in the mouth. A slight discharge of pus from around the affected tooth or teeth might account for this taste and odor, but not of necessity. All these symptoms may occur, as it were, simultaneously, or any two of them may exist without the third. They all belong to the final stages of the trouble, and precede, without treatment, a limited time only the final loss of the teeth affected. It will be noticed that I only allude to a discharge of pus as a probable incident manifesting itself in the later stages, so sure do I feel that it is not a prominent indication to go by. The dentist who looks for it as an aid in his diagnosis, oftentimes will look in vain, and still the disease may be making rapid progress. The presence of pus comes only as a sequence to an active inflammation, and can be counted on only as one of its signs and indications. It does not represent the disease any more than the interest on a debt represents its principal or what it was for.

In the second class of symptoms, those that require both skill and knowledge to discover, belong a much larger group of symptoms, and really the most important for us to consider; for they lie at the very basis of proper diagnosis and treatment, and as prevention is more valuable than either of these, their due consideration is most important. Just here I may say that the terms symptoms and causes seem to run together, and it is hard to decide where the one begins and the other ends. But, for our purpose, we need not be too particular in the use of words so long as they properly indicate our thoughts, and so, if I call a symptom that which appears to any of you a cause, my explanation will be all sufficient to satisfy your scruples.

To the second group of symptoms or indications belong the following: 1. The gum over the affected tooth will have a slightly darker color than is natural, and, may be, will have fallen slightly away from the root; 2. The instrument can be passed up between the gum and teeth beyond the normal distance, which is about one-sixteenth or one-eighth of an inch; 3. The depth will vary at different points more than it ought to; 4. The border of the alveolar process will be felt by an exploring instrument at one or more points; 5. The neck of the tooth will be unduly sensitive, showing the presence of some irritating agent; 6. The neck of the tooth will be rough and uneven under the margin of the gum; 7. A whitish, milky exudation can be pressed from between the gum and the tooth, not at all, however, like pus or simulating it in color or consistency.

To sum them up, a close examination will reveal departures from the normal, healthy condition of the parts under consideration, slight and of apparently little import, but all having a direct and positive bearing on the future health and safety of the tooth. It is well to give special thought to first causes and to be very careful in treating symptoms. To consider them in their natural order and sequence. A neglect in these particulars may lead to grave errors in judgment, and still graver in treatment. Physicians see this now clearly, and ever-increasing thought is being given in all cases to make their practice as scientific as possible, by considering first of all the etiology of the diseases they are called upon to treat. They recognize the truth of the statement that treatment of disease must be empirical, if not based on a clear conception of its cause or causes, and to-day medical literature teems with investigations and studies in this direction. So it must be with us in our specialty, and in that way only can we make our practice certain and sure.

A word, then, first, in reference to the etiology of pyorrhœa alveolaris. To me it has always seemed that much of the trouble that many dentists meet with in their practice arises from a total misconception of its origin and cause; and, again, that this misconception is largely induced by our very ridiculous nomenclature. Anything more absurd from a pathological stand-point than this term "pyorrhœa alveolaris" cannot well be imagined. The literal interpretation of the term is, "A discharge of pus from the alveolar cavity." It is a beautiful example of putting the cart before the horse,—of naming a disease by one of its effects,—and ignoring the cause or causes of its inception. But this is a little bit of folly that dentistry has borrowed from medicine. The full beauty of the term with us may be grasped when we consider, first, that the pus in the majority of cases does not ooze from the alveolus at all; and, second, that the disease may and often does run its course without the formation of any pus whatever. Then, again, we find a genuine discharge of pus where it has its undoubted origin from another cause,—from an alveolar abscess, where the discharge takes place from some point or points around the neck of the tooth. In such a case our nomenclature would be perfect and indicative of the disease; it would be a genuine case of pyorrhœa alveolaris, and yet no one for a moment would be misled and call it by that name. The name, then, would seem to offer a good excuse for some blunderings in practice, as many, indeed, as the different ideas that dentists might hold of the nature of the enemy they were expected to tackle, whose name was only a cloak with which to hide his form and power. The first step, then, I would advise any one to take would be to discard this misleading term entirely; throw it out of his vocabulary, and to approach the subject from the direction of close observation of facts and conditions as he finds them in the mouths of his patients. Without being able to suggest a short, comprehensive title, one handy for use in conversation, I would group all these lesions under the heading of *affections of the peridental membrane, having their origin at the neck of the tooth.*

At the June (1888) meeting of the Philadelphia Odontological Society, I made this assertion for which I was strongly taken to task: "I desire to state positively my belief that pyorrhœa alveolaris is always preceded by a deposit of serumal tartar. Now it is quite possible that this statement will have to be modified, not, however, to a very great extent. The essential principle or thought I wish to leave undisturbed. If I had said that as a rule the so-called pyorrhœa alveolaris had its origin in a purely local cause or causes, and that nine times out of ten this local cause was tartar in some one of its protean forms, I would have rightly stated my opinion. If I had gone farther, and stated that the constitutional diathesis theory was only one way of begging the question and could not stand close examination, I would have still further enforced the same thought. For if I am convinced of any one thing thoroughly, it is that close observation will almost invariably detect the local irritant at the foundation of the trouble, and that treatment based on this theory assures more favorable results than any other with which I am acquainted. Given a primary local source of irritation, and there are many, some of which we will more especially refer to presently, and the rest follows in the natural order of cause and effect. The utmost that can be said for a systemic origin for the disease is that the inflamed gingivæ or mucous membrane is prone to secrete—if that is the proper expression—lime salts, and that these lime salts, in turn, become an added cause of irritation and inflammation to the already affected soft tissues, more especially when they are deposited on the necks of the teeth and below the free margins of the gums. Of course, all will naturally draw a sharp line of distinction between ordinary salivary tartar which is deposited in thick masses on the lingual faces of the lower front teeth or buccal faces of the upper molars, and is seldom or never of itself the cause of pyorrhœa alveolaris, and the various forms of black and brown tartar that creep under the gums and up on the roots of the teeth. The first mechanically pushes the gums back or lengthwise down the roots of the teeth, but does not insinuate itself between the gums and the roots of the teeth. In consistency it is only a semi-solid, and is readily scaled away from the face of the tooth, and, when removed, the soft tissues are found to have been but little affected by its presence; they may bleed a trifle, but practically are in a healthy condition, and soon recover any little loss of tone, once freed from the superincumbent mass. The deposition of this form of tartar may safely be said to be constitutional in that it is not necessarily, or even commonly, preceded by any local inflammation to induce it; but it is not especially dangerous, except through gross neglect, and, as an exciting cause for the disease we are considering, is hardly worth mentioning.

But the black or brown tartar is of another character,—serumic tartar, as Professor Black calls it,—and the name is a good one, as clearly indicative of its origin. It is an exudate from the inner surface of the gingivæ, where they hug the neck of the tooth, an abnormal deposit from the mucous glands in that locality when they are in an irritated or inflamed condition. The physical appearances of this form of tartar vary considerably; but I cannot occupy your time by indicating all of them, the more so as they will naturally suggest themselves to you without such effort on my part. As a rule, it is invisible to the eye except when, having been present for some time, the overlying gum has been destroyed, and so exposed it. It is, therefore, not found on any portion of the crowns of the teeth, the reverse of the case of salivary tartar. For brevity's sake, I will draw your attention to two conditions only. First, when it is found in a comparatively thick mass lying under the free margin of the gum and longitudinally with the neck of the tooth; and, second, where it takes the form of thin scaly patches, having a smooth, hard surface, and clinging most tenaciously to the tooth. The first form is rather the most common of the two, is more easily detected, and, as it does not cling so closely to the tooth, is the most easily removed. The latter—the one having the thin scaly character—I consider by far the most dangerous. It is the most insidious in its nature, and its first effects are slight and insignificant and easily overlooked. Being smooth and hard, it does not irritate the soft tissues, and consequently there are no outside physical manifestations of its presence until its work of destruction is nearly complete. A delicate touch is not enough to prove its existence, for no touch is delicate enough to tell where the root is coated with it and where it is not, so thin is it, so hard, and so like the root itself. As a rule, we can only surmise its presence by one sign only,—viz., when a delicate instrument can be passed between the root and the gum beyond its natural depth, all other conditions being apparently normal. I would draw special attention to this form of tartar, and wish to take great pains to impress on your minds the necessity of fully comprehending its dangerous nature and the extreme care required to diagnosticate its presence, for I am fully convinced that it is frequently overlooked, and that such oversight leads to fatal errors in practice. It is to the failure to detect it that we hear so much about constitutional causes, and the consequent lame excuses for faulty treatment and unfavorable results. Of course, the dentist who does not see a local cause for a local trouble falls back on the body as a whole to explain away his difficulty and father his failures, and the unfortunate patient is made happy and comfortable with the ready explanation that he owes the loss of his teeth to a bad "constitutional diathesis," and "that nothing can be done for it," and he takes away his sound tooth, after it is extracted, showing only a little discoloration that may be on the roots, to prove the wisdom of his dentist and his unfortunate relations with an all-wise Providence. The medical doctor looks wise and a bit sad as, with a twinkle in one corner of his eye, he tells his patient he has malaria, and that he cannot hope for much, for his system is full of it, and the dentist in like manner folds his hands and says, "Poor fellow! It is constitutional, and I can do nothing for you. Hold on to them as long as you can, and then I will take them out and make you a nice gold plate with artificial teeth, and you will never know your loss. You don't know, my dear sir, what wonderful strides dental science has made in late years, and how skilfully I can make good your misfortune." If dentist and patient would both take those poor discolored roots in their hands, and give them a careful examination, their eyes might be opened very wide, and they might both exclaim, but with widely different thoughts and feelings, "Is it possible!" The dentist, with his excavator, would find that the discolored patch could be chipped off, thin as it was, and that the healthy root was immediately underneath, and the thought would suggest itself that a foreign body of that character, between the root and the peridental membrane, was not conducive to the health and comfort of his patient; and if it had been removed in time, and in the mouth, and not out of it, might have prevented its loss, and have given him a better claim to the title, "a skilful practitioner." As to the thoughts that would worry the patient, it may well be deemed prudent to say as little as possible. The simple truth is that the various troubles that teeth are heir to have no remote origin. They are so purely local and so close to hand that it seems unpardonable to overlook them. Do not misunderstand me on this point. I fully appreciate the importance of having in mind systemic conditions and influences, and their direct and indirect bearings in diagnosis and treatment, and give full weight to it all, but I do not allow my mind for a moment to be withdrawn from the main fact, that I have to deal with a local disease produced by local causes, and that the state or condition of the system is only one factor to be considered, and not the immediate or direct one.

If the tartar deposit could be eliminated from the list of causes of the disease in question, the disease itself would practically disappear, so manifestly is it the prime cause of the disease and so little have other causes to do with it. Nine-tenths, if not ninety-nine-hundredths, of the cases that present themselves are due to it. Still there are other ones to be considered, and we will briefly allude to them, but only in a general way. They may be grouped together under the general heading of mechanical or chemical irritants, foreign to the oral cavity, and accidental in their presence. A plate unduly pressing on the neck of the tooth may be one cause; a bristle from a tooth-brush or other foreign body lodged under the margin of the gum and pressing on the membrane, another; putrefying food allowed to remain in contact with the tooth and generating some poisonous ptomaines which gain lodgement at the gingival border, another; and so the list might be multiplied, but we will hasten on.

And now, for a moment, let us see if we cannot go a step farther back in seeking for the origin of the trouble. Is there not a first cause that should be considered one of equal, if not of greater, value than any of those we have already alluded to? I think there is. If I am right in my theory, the healthy mucous glands of the gingivæ do not secrete tartar. It is an abnormal secretion from glands in an unhealthy condition. Now, the question arises, What is it that destroys the healthy action of these glands and makes them a source of danger? If we can answer this question, we have gone a long way in the solution of our difficulty. And just here I hesitate somewhat, for I do not feel quite sure of my position, but I am anxious to place the thought before you in hopes, at least, of exciting criticism, and obtaining new light where I am so much in doubt.

I am strongly, then, of the opinion that a natural or acquired roughness of the neck of the tooth, under the free margin of the gum, is the main cause we are seeking. That such a roughness is far more common than is generally supposed I am fully convinced, and also that it is no forced conclusion to consider it a source of irritation to the open mouths of the glands in contact with it. It is quite possible likewise that just here the *rôle* of micro-organisms may be most important to consider, and that the poisonous ptomaines they produce, in growing, may have a most injurious effect. Unfortunately, direct experiment in this direction is most difficult to carry out, and we can only reason in an indirect way; but the field is a most inviting one for research and thought, and offers vast possibilities in the way of treatment. Of one thing I feel quite confident, and this I say from the light of many years' practice, that if the neck of the tooth, under the margin of the gum, is kept clean and polished, no tartar of any description will gain a lodgement upon it.

There is but one meaning to all that I have said so far in its relation to treatment. Taking the ground I do, that a local irritant is the prime cause of the trouble, and that constitutional conditions only indirectly modify or influence its character and duration, all treatment must be based directly in the line of removing this irritant, and then in bringing the parts affected into a healthy normal state. The mode of procedure should be precisely analogous to that in surgery, which requires the removal of a splinter from the flesh or foreign body from a wound, as a preliminary to bringing about a healthy condition of the affected parts. Nothing more and nothing less is required.

First, then, in order of consideration, we have the mechanical removal of the tartar, or other exciting causes, by means of instruments specially devised for that purpose. In the use of instruments, though, the personal equation plays an important part, and instruments and methods will vary with the mental and physical peculiarities of each individual. All meet on common ground, though, in a few essentials, first among which I would say to be the complete removal of the tartar with as little injury to the soft parts as possible. For this purpose Dr. Cushing devised a set of instruments to be used on the push-principle, and Dr. Black strongly endorses them. My objection to them is twofold: First, they fail to reach all portions of the tooth to be operated on; and, second, their use endangers the soft parts; furthermore, the push motion, which separates the tartar from the root, does not remove it from the pocket, but leaves it there to be a further source of irritation. The force required to separate the tartar from the tooth, at times, is very considerable, and it is difficult to stop the instrument in time. Still, I would not be without them, or others of similar character, but I could not rely on them altogether. Those I prefer work on the principle of the hoe or scraper, and most of my work is done with them. The forms or shapes are almost numberless; and there is far more danger in not having variety enough than in having an excess. So much of success depends on readily reaching every portion of the coated tooth. To reach around and between roots —especially molar—requires many apparently eccentric shapes. Each operator, though, will be able to study them out for himself. Some of those I most commonly use I have brought with me, and will be happy to show to any of you.

But the steel, no matter how cunningly fashioned, will oftentimes fail to reach all parts, and we must seek other means to accomplish our ends; and no other is available that I know of except an acid, and this I constantly make use of. It takes very little acid to soften any form of tartar sufficiently so that it will wash off by a strong stream of water from a syringe or can be wiped off with a small pledget of cotton wrapped around a platinum point. It is a mistake to use the acid as liberally as many do. A few minims of an eight- or ten-per-cent. solution of sulphuric acid, following the scraper or hoe, will generally suffice; but if more is required,—and it may be,—the applications should be twenty-four or forty-eight hours apart. I employ the chemically pure acid, and dilute it myself. The aromatic sulphuric acid I long since discarded. Instead of the acid, peroxide of hydrogen may be employed to good advantage, its solvent action on the tartar being nearly equal to the acid. In fact, just here it acts like an acid.

Cases will frequently present themselves where a single tooth will be badly affected, so much so as to be very loose and shaky, and yet a good portion of the root-membrane and socket be in a fairly healthy condition. Of course, the constant motion of the tooth tends to still further break up its attachments and hasten its loss. A simple device which I have frequently employed is here most serviceable. It is a device which, though I do not know of any other dentist using it, meets so directly the necessities of the case, that I take it for granted that it is by no means new or peculiar to my practice. I refer to a brace so made as to make the neighboring firm teeth support and hold in place the loose one. The manner of making them will readily suggest itself to you all. They should be so constructed that the patient can easily remove them and put them in place. The fit should be a good one, and great care should be taken in its adaptation. A patient who has once worn one of these braces will never, so long as the tooth remains in his head do without it,—so great is the comfort and confidence it gives them. Even where there is a vacant space next the loose tooth a satisfactory brace can be made, the vacant space being filled in in such a manner as to prevent the food crowding under it.

Sometimes it will happen that one root will be so badly affected —as in the case of molars—that it will be impossible to save it, and it will become a source of danger to the remaining sound ones. Amputation of the offending part is here clearly indicated, and should be resorted to without delay. For this purpose I generally use the engine, armed with a sharp fissure-burr. In this way it can easily be cut off, and without shock to the balance of the tooth.

Let me here draw your attention to the fact that when the disease attacks pulpless teeth, full success in treatment can seldom, or never, be hoped for, and, at the best, the actual loss of the tooth can only be retarded. Sooner or later it is pretty sure to go. The reason is manifest on a little thought. The peridental membrane has a twofold source of vitality. The nerves and capillaries going to nourish and sustain it enter it from opposite directions,—from in and around the gingival border and from its apical end. Either source of supply is sufficient of itself to keep the membrane in a fairly healthy condition for a long time; but if both are affected or destroyed, the intervening membrane soon loses its vitality and becomes necrosed. There is seldom much pain accompanying this death of the peridentium. As it dies it seems to disintegrate, and dissolves out; and when the tooth is extracted, not a particle of the membrane will be found adherent to the root, but it will present a clean and polished appearance.

The age of the patient, the duration of the disease, and the general condition of the patient have to be all considered in treatment. If, from any cause the parts have lost a portion of their vitality, and do not respond quickly, or not at all, to treatment, then the case becomes more or less hopeless. As a rule, we can but assist nature in bringing about a cure, first by removing the exciting cause of the trouble, and then by such tonic applications as may bring new life to the tissues enfeebled by age or disease. All this portion of my subject I must beg leave to omit, so great is the diversity of opinion as to their relative values and their specific action. For myself, there are but few to which I attach any special value; and if nature, unaided, or assisted only by giving her a fair chance, cannot bring about the desired ends, I do not expect much help from local applications.

To illustrate one phase of pyorrhœa alveolaris, I have brought

Fig. 1.

casts of a case with me. In June, 1874, the teeth were perfectly regular in the mouth of a gentleman aged thirty-five or thirty-six, and also in a healthy condition. Soon after that, he felt the front teeth pressing tightly together; the three front teeth commenced to crowd each other; and in the autumn of the same year, and during the winter, the left central pushed against the right central, and finally commenced to overlap it. (See Fig. 1.) In the spring of 1875 the mouth was very much in the condition as that shown by Figure 2, which is a front view, and does not exaggerate the deformity in the least. He came to me in 1887. The history of this case was precisely such as those described in my paper, in that he had been told that the trouble was constitutional, in the system; and nothing could be done; and so the disease was allowed to progress. On examination I found a pocket on the lingual face of the right central, extending nearly to the apex of the root. On the left central the pocket had extended quite to the apex of the root, and had cut off the pulp where it entered the apical foramen, and the lateral was dead. A very careful examination, which I made by putting some cotton in the pocket two or three days in succession and pressing the gum back, enabled me to see a dark *shade*, indicating serumal tartar of the scaly character that I have described. The thickening of the membrane on the inner surface of the tooth had crowded it to the right and outward. If that pocket had been discovered in time, and the thin scale of serumal tartar had been removed, this deplorable condition of affairs never would have been brought about, and he would have had his teeth in a sound and healthy condition to-day.

Fig. 2.

The treatment consisted, first, in the use of scalers to remove the tartar, followed by the application of peroxide of hydrogen. For a month or six weeks the teeth were treated once or twice a week. At the end of that time the teeth which were quite loose when I commenced the treatment—the lateral being very loose—were in a much firmer condition, showing that the tumefaction of the peridental membrane had been very much bettered. When I had succeeded in removing the tartar and quieting the inflammation, I commenced to move the tooth around to the left, and to pull it in. The next cast shows the condition of the mouth when the movement of *this* tooth was completed. (See Fig. 3.) You see it has been pushed out of the socket to one-eighth of an inch. For two months I tried, by various methods, to push it up in the jaw, but with very little success; and I was finally compelled to shorten it by grinding off the cutting edge. I treated the lateral by removing the dead pulp and filling the pulp-chamber. The last cut (see Fig. 4) shows the condition of the teeth at the present time. This left central, which was at the commencement of the operation quite loose and out of place, is now almost as firm as the right central, and there is every indication that the disease is completely cured.

FIG. 3.

He is now wearing a stay-plate, as in an ordinary case. I have here a bottle of teeth, showing all the varieties of serumal tartar that I spoke of. Notice, especially, the two bicuspids, showing the scaly tartar. The teeth look as though they had a slight coating of varnish.

This case merely illustrates the effect of serumal tartar, of the thin and scaly character, very beautifully. But, after all is said and done, it is not more instructive than others that are occurring

FIG. 4.

constantly in practice, but it is more showy; and that is the reason, more than any other, that I have brought it here. If a sound and firm tooth in the mouth of a man of thirty-six years can be pushed completely out of its place inside of eight months by swelling of the peridental membrane, induced by tartar, it shows the necessity of an early and correct diagnosis and prompt and thorough treatment. There is no new deposit of bone whatever. The parts around the tooth have assumed a normal condition, and the pocket remains as it was when I commenced.

## Figures and Tables

**Figure f1:**
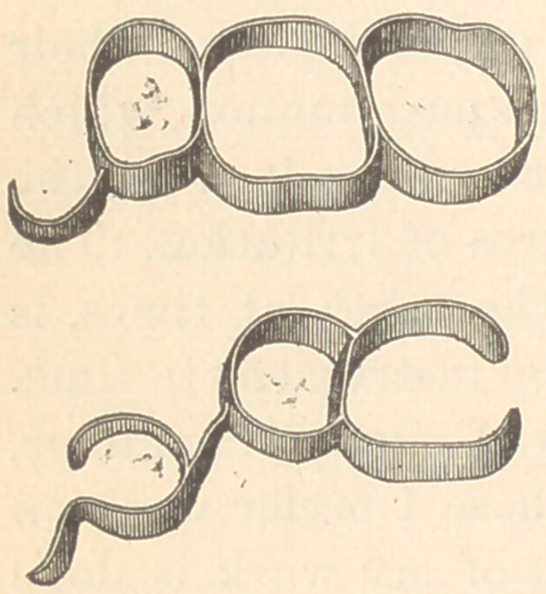


**Fig. 1. f2:**
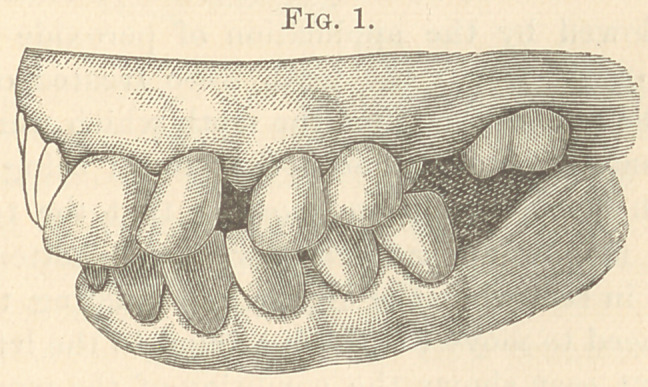


**Fig. 2. f3:**
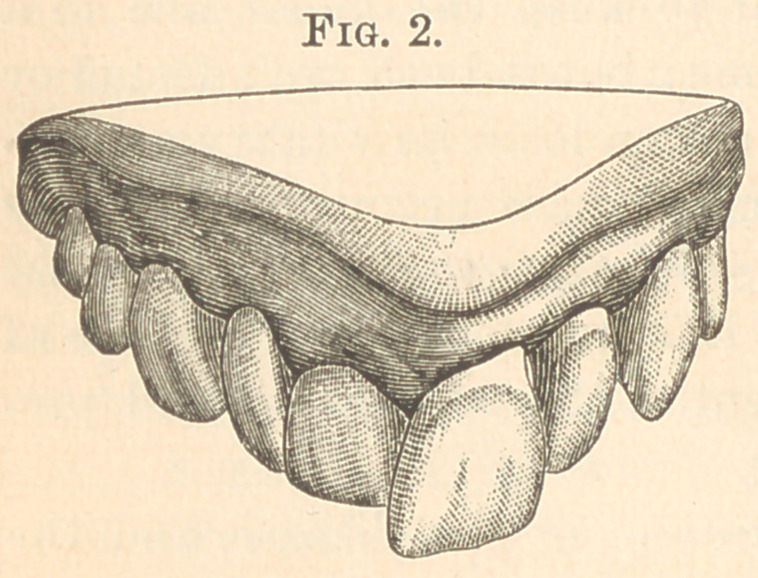


**Fig. 3. f4:**
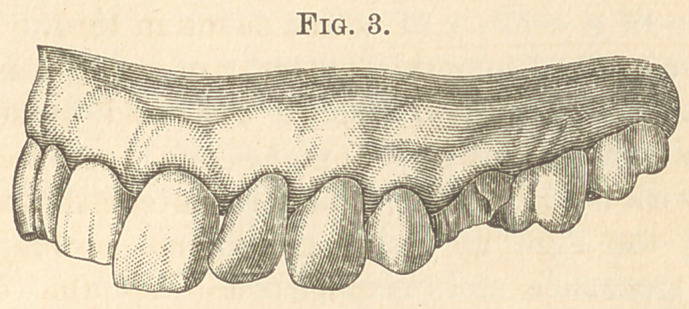


**Fig. 4. f5:**